# MiR-657/ATF2 Signaling Pathway Has a Critical Role in *Spatholobus suberectus* Dunn Extract-Induced Apoptosis in U266 and U937 Cells

**DOI:** 10.3390/cancers11020150

**Published:** 2019-01-28

**Authors:** Hyun Ji Lim, Moon Nyeo Park, Changmin Kim, Beomku Kang, Hyo-Sook Song, Hyemin Lee, Sung-Hoon Kim, Bum-Sang Shim, Bonglee Kim

**Affiliations:** 1 College of Korean Medicine, Graduate School, Kyung Hee University, Seoul 130-701, Korea; hyunjilim@khu.ac.kr (H.J.L.); mnpark@khu.ac.kr (M.N.P.); glansy555@gmail.com (H.L.); sungkim7@khu.ac.kr (S.-H.K.); eshimbs@khu.ac.kr (B.-S.S.); 2 College of Korean Medicine, Kyung Hee University, Seoul 130-701, Korea; ckdals4302@khu.ac.kr (C.K.); beomkukang@khu.ac.kr (B.K.); 3 Department of Science in Korean Medicine, College of Korean Medicine, Graduate School, Kyung Hee University, Seoul 130-701, Korea; shs331@khu.ac.kr

**Keywords:** *Spatholobus suberectus* Dunn, multiple myeloma, myeloid leukemia, reactive oxygen species, *N*-Acetyl-L-cysteine, endoplasmic reticulum stress, activating transcription factor 2, C/EBP homologous protein, apoptosis, miR-657

## Abstract

Though *Spatholobus suberectus* Dunn (SSD) has been reported to have anti-virus, anti-osteoclastogenesis, and anti-inflammation activities, its underlying anti-cancer mechanism has never been elucidated in association with the role of miR-657 in endoplasmic reticulum (ER) stress-related apoptosis to date. SSD treatment exerted cytotoxicity in U266 and U937 cells in a dose-dependent manner. Also, apoptosis-related proteins such as PARP, procaspase-3, and Bax were regulated by SSD treatment. Furthermore, Terminal deoxynucleotidyl transferase dUTP nick end labeling (TUNEL) assay revealed that a number of apoptotic bodies were increased by SSD. Interestingly, the ER stress-related proteins such as p-ATF2 and CHOP were elevated by SSD. Interestingly, reactive oxygen species (ROS) generation and cytotoxicity by SSD treatment were significantly reduced by *N*-Acetyl-L-cysteine (NAC). Among the microRNAs (miRNAs) regulated by SSD treatment, miR-657 was most significantly reduced by SSD treatment. However, an miR-657 mimic reversed SSD-induced apoptosis by the attenuation of the expression of p-ATF2, CHOP, and PARP cleavage. Overall, these findings provide scientific evidence that miR657 is an onco-miRNA targeting the ER stress signal pathway and SSD induces apoptosis via the inhibition of miR-657, ROS, and the activation of p-ATF2 and CHOP as a potent anti-cancer agent for myeloid-originated hematological cancer.

## 1. Introduction

Myeloid-originated hematological malignancies, including multiple myeloma (MM) and myeloid leukemia (ML), have been constantly increasing worldwide [1,2]. MM is a tumor of long-lived malignant plasma cells in the bone marrow, leading to infection, osteolytic bone lesions, hypercalcemia, or renal insufficiency [3]. ML is the abnormal differentiation of myeloid cells in the bone marrow, leading to fatigue, hemorrhage, infection, and organ infiltration, such as hepatomegaly and splenomegaly [4,5]. Myeloid-originated hematological malignancies are capable of escaping programmed cell death, triggering proliferation and metastasis. Therefore, the induction of apoptosis has been considered as a target for cancer treatment [6,7]. Recently endoplasmic reticulum (ER) stress has been suggested to mediate apoptosis in cancer cells [8,9]. Moreover ER stress-mediated apoptosis in myeloid-originated hematological malignancies has been proven [10,11].

The key mechanism in this study is the apoptotic mechanism by miR-657 and reactive oxygen species (ROS) generation-mediated ER stress. ER stress is initiated by various causes, such as glucose deprivation, hypoxia, and redox perturbation [12]. These cause the increase of unfolded proteins in the ER. This usually leads to the initiation of ER stress-related proteins, which are known to be associated with the induction of apoptosis in cancer [13]. Mainly three ER transmembrane proteins take lead in the process, which are (1) inositol-requiring enzyme-1 (IRE1), (2) activating transcription factor 6 (ATF6), and (3) PKR-like ER kinase (PERK). These proteins mainly respond to the accumulation of unfolded proteins, and the following is a brief description of each protein. IRE1α consists of an *N*-terminal luminal sensor domain, a single transmembrane domain, and a *C*-terminal cytosolic effector. IRE1α oligomerization is induced by the accumulation of the unfolded proteins in ER membranes and the auto-phosphorylation of the cytosolic domain of IRE1α [12]. Increased unfolded protein response (UPR) activates the three proteins mentioned above in the ER, inducing the inhibition of protein entry in to the ER by arresting mRNA translation and increasing the expression of ER chaperones. Then, ER stress signaling transduction pathways are activated, leading to cell death mechanisms under certain condition [14,15]. ATF6 is a mammalian transcriptional factor that mediates ER stress [16]. ATF6 cleavage is usually induced by the accumulation of protein in the ER. Following its cleavage, its cytoplasmic domain is released into the nucleus [17]. When the unfolded proteins in a cell accumulate, it results in the unfolded protein response (UPR) [18]. PERK is also a major factor in ER stress, and its downstream target, C/EBP-homologous protein (CHOP), is reported to stimulate programmed cell death—apoptosis—by promoting protein synthesis and oxidation [19]. Oxidative stress is a condition in which the balance of antioxidant and ROS production is disturbed [20]. ROS overproduction causes oxidative damage and affects ER homeostasis, inducing the interference of protein folding and ER stress. Long-term ER stress induces CHOP, activating other pro-apoptotic factors including caspases and PARP [21]. These are the main pathways of this study, specifically focusing on PERK, which is upstream of eIF2α, ATF4, and CHOP. Furthermore, UPR has been identified to be linked with microRNAs (miRNA or miR), which consist of 20–22 single-stranded RNAs that regulate gene expression in a sequence-specific manner [22,23]. Evidence has shown that the inhibition of miRNA may lead to the progression of cancer growth [24,25,26]. Among the many miRNAs, miR-657 is known to be an onco-miRNA in cancer [27]. However, the role of miR-657 in ER stress has not yet been reported. The agent used in this study, *Spatholobus suberectus* Dunn (SSD), has been reported to have anti-oxidant [28], apoptosis-inducing [29], and cell cycle arrest-inducing activities [30]; however, its miR-657/ER stress-mediated apoptotic mechanism is not well-studied. In this study, we reveal a novel anti-cancer mechanism of SSD via miR-657/ER stress-mediated apoptosis for the first time.

## 2. Results

### 2.1. Identification of Epigallocatechin (EGC) and Genistein from SSD through HPLC Analysis

**Figure 1:**
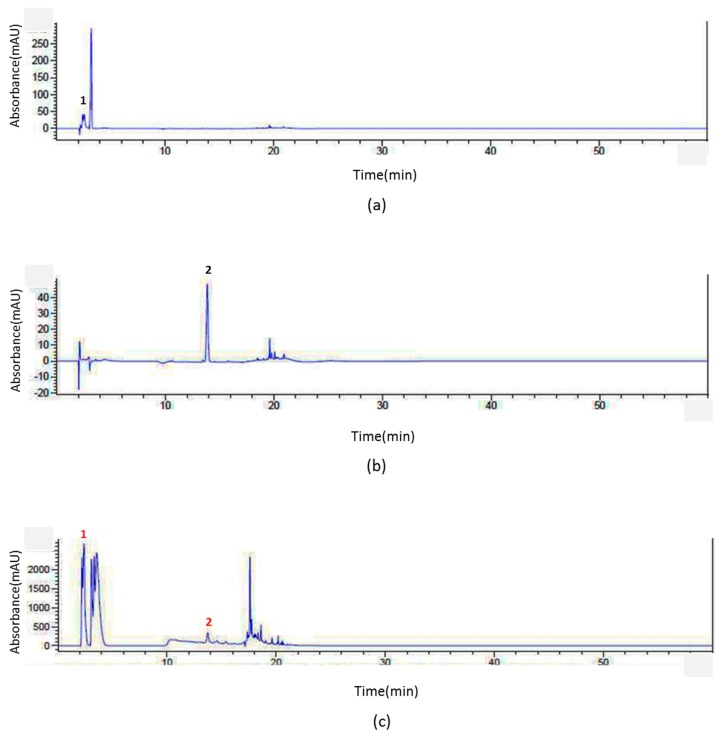
HPLC analysis of *Spatholobus suberectus* Dunn (SSD). In order to obtain efficient chromatographic methods for the analysis of (**c**) SSD compared with (**a**) epigallocatechin (EGC) or (**b**) genistein in accordance with the retention time and UV-Vis wavelength, an Agilent series 1290 system was used. To exhibit efficient separation and reasonable results, intra-day and the inter-day analyses were conducted in the same day and on consecutive three days, respectively. The gradient eluent condition resulted in a satisfactory separation. The relative standard derivations (RSDs) were deliberated to indicate the level of precision.

To confirm that an extract contains constituents of SSD, HPLC analysis was conducted. Among many of the constituents of SSD, epigallocatechin (EGC) and genistein were tested [31]. The standard peaks 1 and 2 were identified as EGC (Figure 1a) and genistein (Figure 1b) according to the retention time (RT) and UV-vis spectra of the standards. The results showed that the extract contained those two compounds, EGC and genistein. The repeatability and reproducibility were verified by triplicate analyses. Both the intra-day and the inter-day relative standard derivations (RSDs) were less than 0.9% (Table 1). The percentages of EGC and genistein in the SSD extract were 1.186% and 3.54%, respectively.

### 2.2. SSD Exerted Cytotoxicity in Hematological Cancers but Less in Normal Cells

**Figure 2:**
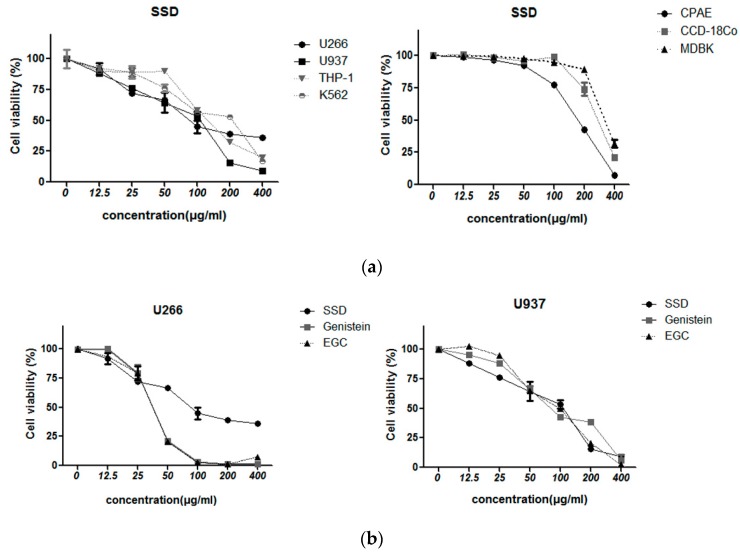

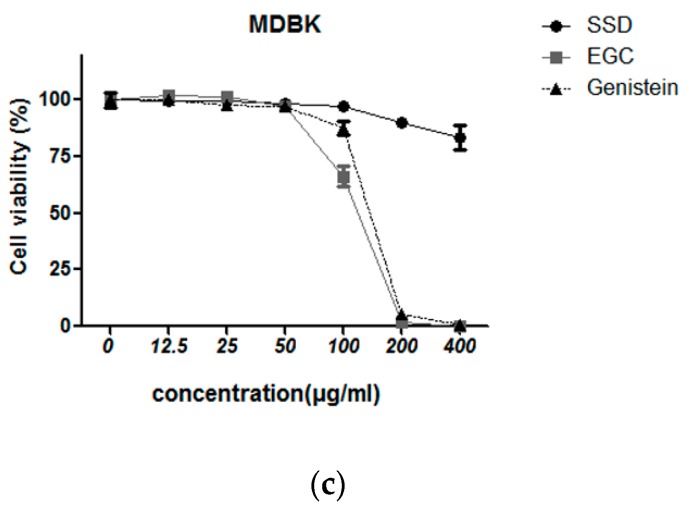
The cytotoxic effect of SSD. (**a**) U266, U937, THP-1, K562, CPAE, CCD-18Co, and MDBK cells were seeded into 96-well microplates and treated with the indicated concentrations of SSD for 24 h (sample number *n* = 3). (**b**) U266 and U937 cells were seeded into 96-well microplates and the indicated concentrations of SSD, EGC, and genistein were added for 24 h (sample number *n* = 3). EZ-CYTOX assay was used to measure cell viability. (**c**) MDBK cells were exposed to 0, 12.5, 25, 50, 100, 200, and 400 µg/mL of SSD, EGC, and genistein for 24 h (sample number *n* = 3). Results show a representative of three independent experiments. Results are presented as the means ± SD.

To evaluate the cytotoxic effect of SSD against hematological cancers, EZ-CYTOX cell viability assay was performed. Cells were treated with various concentrations (0, 12.5, 25, 50, 100, 200, 400 µg/mL) of SSD for 24 h. SSD exerted significant cytotoxicity in cancer cells, including U266, U937, THP-1, and K562 cells, as well as in normal cells, including CPAE, CCD-18Co, and MDBK cells. SSD treatment exerted cytotoxicity in multiple myeloma U266 cells and myeloid leukemia U937, THP-1, K562 cells. However, CPAE cells (normal pulmonary artery endothelial cells), CCD-18Co cells (normal colon epithelial cells), and MDBK cells (normal kidney cells) were not affected by up to 50 µg/mL of SSD treatment (Figure 2a). The doses of SSD used in this study were 10 and 20 µg/mL, which are toxic to hematological cancer cells but harmless to normal cells. To compare the cytotoxic effect of SSD and its components, genistein and EGC were also administered to U266, U937, and MDBK cells. As shown in Figure 2b, genistein or EGC tends to exert higher cytotoxicity in U266 from 25 µg/mL and similar cytotoxicity in U937 cells. However, up to 25 µg/mL the cytotoxicity of SSD was similar or even higher than that of genistein and EGC. Also, in the normal MDBK cells, genistein and EGC showed higher cytotoxicity compared to SSD nearly from 50 µg/mL (Figure 2c).

### 2.3. SSD Induced Apoptosis in U266 and U937 Cells

**Figure 3:**
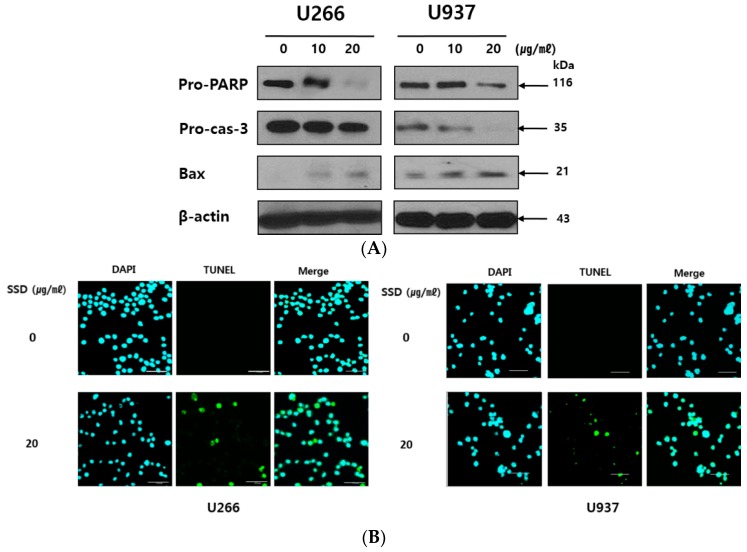
Effect of SSD on apoptosis-related proteins and transferase dUTP nick end labeling (TUNEL) -positive cell population in U266 and U937 cells. (**A**) Cells were exposed to SSD (10 or 20 μg/mL) for 24 h. The lysates were subjected to Western blot analysis for pro-PARP, pro-caspase-3, Bax, and β-actin. (**B**) Cells were treated with 20 μg/mL of SSD and used for TdT-mediated dUTP nick end labeling (TUNEL) staining (sample number *n* = 3). Scale bar: 50 µm. Cells were visualized using an Olympus FLUOVIEW FV10i confocal microscope. Results show a representative of three independent experiments.

To identify whether the cytotoxic effect of SSD was due to apoptosis induction, U266 and U937 cells were treated with 10 and 20 µg/mL of SSD for Western blot analysis and transferase dUTP nick end labeling (TUNEL) assay. As shown in Figure 3A, SSD cleaved PARP and capase-3. Bax, a pro-apoptotic Bcl-2 family, was increased by SSD treatment. Also, TUNEL-positive cells were significantly increased in SSD-treated U266 and U937 cells (Figure 3B). Along with the Western blot analysis results of apoptosis-related proteins, TUNEL assay data revealed the apoptotic effect of SSD in U266 and U937 cells.

### 2.4. SSD Upregulated ER Stress-Related Proteins in U266 and U937 Cells

**Figure 4:**
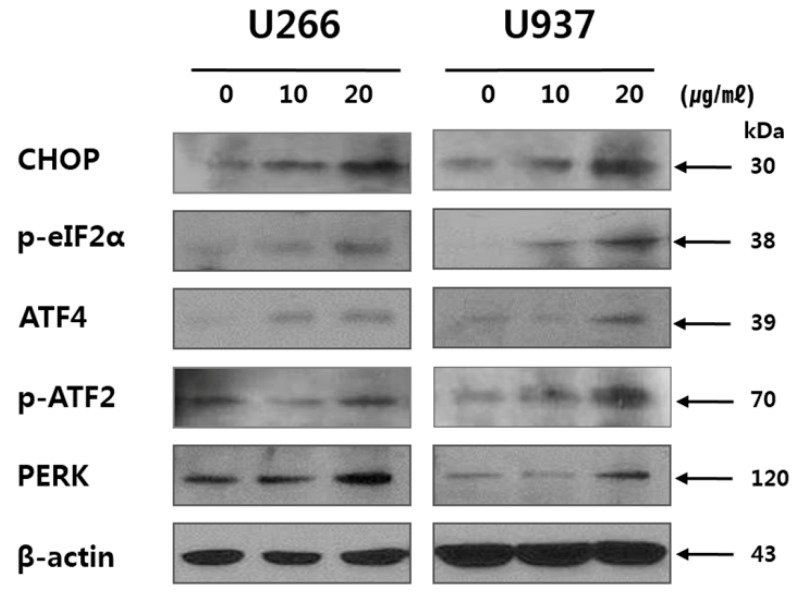
Effect of SSD on endoplasmic reticulum (ER) stress-related protein in U266 and U937 cells. Cells were treated with SSD (10 or 20 μg/mL) for 24 h. The lysates were subjected to Western blot analysis for CHOP, *p*-elF2α, ATF4, *p*-ATF2, PERK, and β-actin. Results show a representative of three independent experiments.

Next, to reveal whether the apoptosis induction by SSD is associated with ER stress, Western blotting for ER stress-related proteins such as CHOP, *p*-eIF2α, ATF4, *p*-ATF2, and PERK was conducted. The result showed that SSD treatment upregulated the expression of ER stress proteins such as CHOP, *p*-eIF2α, ATF4, *p*-ATF2, and PERK in U266 and U937 cells (Figure 4).

### 2.5. SSD Increased ROS Generation in U266 and U937 Cells

**Figure 5:**
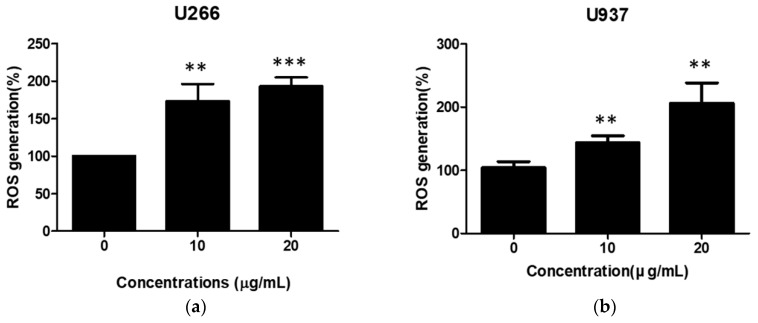
Effect of SSD on reactive oxygen species (ROS) generation in U266 and U937 cells. U266 (**a**) and U937 (**b**) cells were exposed to SSD (10 or 20 µg/mL) for 24 h (sample number *n* = 3). ROS production was analyzed using 2′,7′-Dichlorofluorescein diacetate (DCFDA) by cellular reactive oxygen species detection assay kit. Results are presented as the means ± SD of three independent experiments. ** p < 0.01; *** p < 0.001 versus the control group.

ROS has pivotal role in ER stress-related apoptosis induction [32]. To confirm the effect of SSD on ROS generation, ROS was quantified by 2′,7′-Dichlorofluorescein diacetate (DCFDA) cellular ROS detection assay in SSD-treated hematologic cancer cells. As shown in Figure 5, SSD treatment significantly increased ROS generation in both U266 and U937 cells in a dose-dependent manner.

### 2.6. ROS Scavenger Reversed SSD-Induced Apoptosis in U266 and U937 Cells

**Figure 6:**
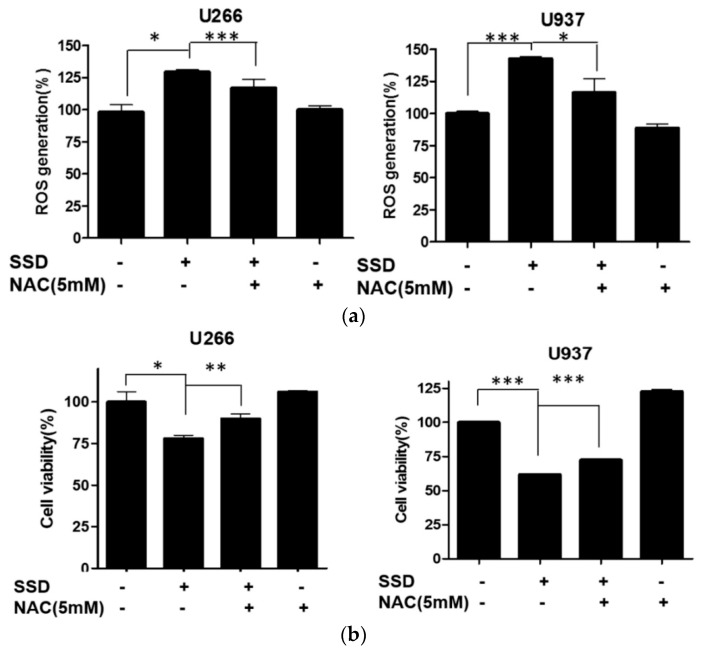
Effect of ROS scavenger on SSD-induced apoptosis in U266 and U937 cells. (**a**) Cells were treated with *N*-Acetyl-L-cysteine (NAC) for 2 h prior to the exposure to SSD (10 or 20 µg/mL) for 24 h. ROS production was analyzed using oxidation sensitive fluorescent dye (DCFDA) by a cellular reactive oxygen species detection assay kit. (**b**) Cells were seeded into 96-well microplates and pre-exposed to NAC (5 mM) for 2 h, then treated with the indicated concentrations of SSD for 24 h. EZ-CYTOX assay was used to measure cell viability in U266 and U937 cells (sample number *n* = 3). Results are presented as the means ± SD of three independent experiments. * *p* < 0.05; ** p < 0.01; *** p < 0.001 between the two groups.

To confirm that ROS generation is associated with SSD-induced apoptosis, an ROS measurement assay and a cytotoxicity assay were conducted with *N*-Acetyl-L-cysteine (NAC)-treated cells. As shown in Figure 6a, increased ROS generation by SSD treatment was significantly reduced by NAC pretreatment. Also, the cytotoxic effect of SSD was significantly downregulated by NAC pretreatment in both U266 and U937 cells (Figure 6b).

### 2.7. SSD Reduced the Expression of miR-657 in U266 and U937 Cells

**Figure 7:**
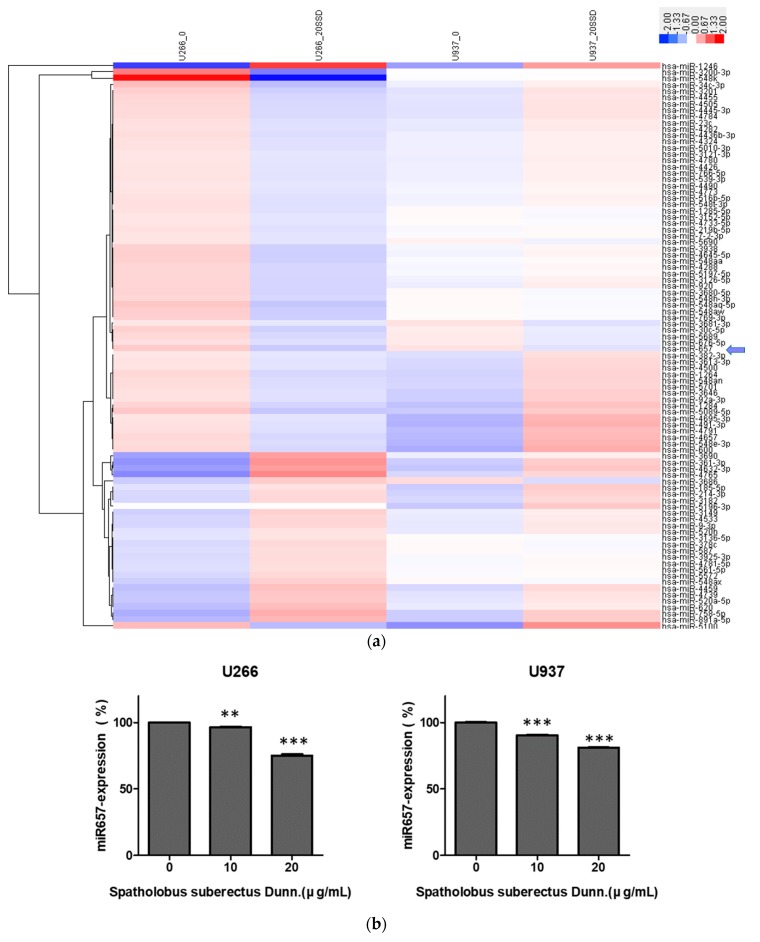
Effect of SSD on microRNA (miRNA) alteration in U266 and U937 cells. (**a**) Heat map clustering of 100 miRNAs involved in ER stress and apoptosis. Red and blue colors represent the magnitude of expression. (**b**) qRT-PCR analyses were conducted for miR-657 with SSD (10 or 20 µg/mL)-treated U266 and U937 cells. Values represent the means of three experiments ± SD; ** p < 0.01; *** p < 0.001 versus the control group.

To elucidate the role of miRNAs in SSD-induced apoptosis, microRNA array and qRT-PCR were conducted. The miRNAs array included a set of miRNAs associated with ER stress and apoptosis. miRNA expression data were obtained and their altered expression was visualized by a heat map (Figure 7a). The results revealed significantly altered expression levels of various miRNAs (*p* < 0.05). Among the miRNAs, the expression of miR-657 was significantly reduced by SSD treatment in both U266 and U937 cells, which is reported to have role in tumorigenesis [27]. To confirm the negative regulation of SSD on miR-657, qRT-PCR for miR-657 was performed. As shown in Figure 7b, the expression of miR-657 in SSD-treated U266 and U937 cells was decreased compared to untreated control groups.

### 2.8. MiR-657 Plays a Role in SSD-Induced Apoptosis in U266 and U937 Cells

**Figure 8:**
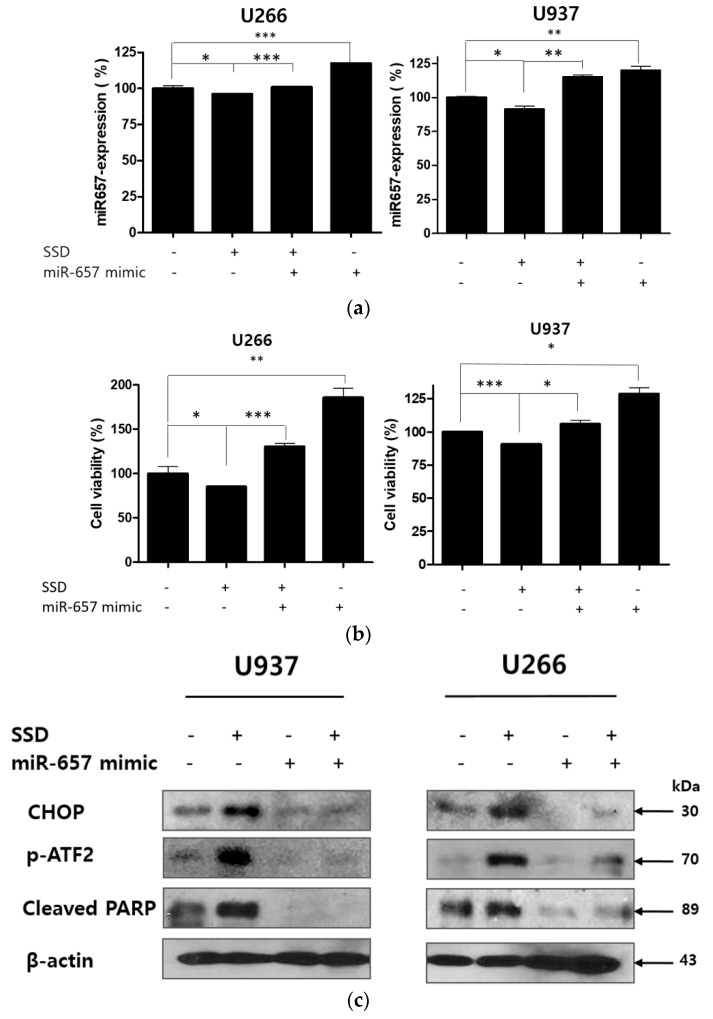
Effect of miR-657 in SSD-induced apoptosis in U266 and U937 cells. Cells were transfected with miR-657 mimic for 48 h (sample number *n* = 4). (**a**) To determine the expression level of miR-657, qRT-PCR for miR-657 expression was performed after 24 h of SSD (20 µg/mL) treatment with or without miR-657 transfection. (**b**) Cell viability was determined by an EZ-CYTOX cell viability assay kit (20 μg/mL of SSD exposure for 24 h). (**c**) Cells were transfected with miR-657 mimic and treated with 20 µg/mL of SSD for 24 h. Western blot analysis was implemented for CHOP, *p*-ATF2, and cleaved PARP. Values represent the means of three experiments ± SD; * *p* < 0.05; ** p < 0.01; *** p < 0.001 between the two groups.

To reveal the further role of miR-657 in SSD-induced apoptosis, U266 and U937 cells were transfected with an miR-657 mimic for 48 h and exposed to 20 μg/mL of SSD. Subsequently, qRT-PCR, viability assay, and Western blot analysis were implemented. As shown in Figure 8a, reduced expression of miR-657 by SSD treatment was reversed in the miR-657 mimic-transfected cells. Also, significantly downregulated cell viability was increased in the miR-657 mimic-transfected cells. MiR-657 mimic-transfected cells showed a significant increase in cell viability compared to the control group (Figure 8b). Western blot analysis data also revealed that the increased expression of p-ATF2, CHOP, and cleaved PARP induced by SSD treatment was attenuated in miR-657 mimic-transfected cells (Figure 8c).

## 3. Discussion

MM is the most advanced plasma cell disease. MM is characterized by the excessive monoclonal proliferation of plasma cells, which secrete monoclonal myeloma proteins (M-proteins) composed of two heavy polypeptide chains of the same class and two light polypeptide chains of the same type [33]. ML is a cancer of the myeloid line of blood cells, characterized by a marrow stem cell disorder in which the accumulation of immature granulocytes such as neutrophils, eosinophils, and basophils is found [34].

Due to the development of proteasome inhibitors (PIs) including Bortezomib and Carfilzomib as well as immunomodulatory drugs (IMiDs) including Elotuzumab and Lorvotuzumab, the median survival rate of patients with MM or ML has been improved [35,36]. However, most patients with MM experience relapse and treatment with anti-hematologic cancer agents leads to side effects such as peripheral neuropathy [36,37]. Patients with ML suffer from anemia as well as easy bruising or bleeding. Moreover, older patients who are unable to receive intensive chemotherapy have a typical survival of 5–10 months [38].

Currently, MM and ML are considered incurable diseases. As such, new approaches using novel materials and mechanisms are needed. Thus, in this study, a new anti-cancer mechanism of SSD against hematological cancer was first elucidated, regarding ROS/ER stress and miRNA regulation. SSD significantly exerted cytotoxicity in a dose-dependent manner in U266 and U937 hematologic cancer cells. The cytotoxic effect of SSD leading to cell death was examined by cytotoxic assay, Western blot analysis, TUNEL assay, and qRT-PCR—all of which demonstrated that various factors that are correlated to ER stress influence the apoptosis mechanism. Of note, PARP, caspase-3 cleavage, and the difference in the level of Bax protein were observed. These results indicate that SSD displays apoptotic effects. Apoptosis is defense mechanism that is employed, for example, in immune reactions when cells are damaged by disease or toxic agents [39]. PARP is a family of proteins involved in the repair of single-strand DNA breaks, subsequently shown to be cleaved into 89- and 24-kDa fragments during drug-induced apoptosis in a variety of cells [40]. Such cleavage disables its ability to respond to DNA strand breaks and inactivates the enzyme [41]. SSD treatment cleaved PARP in both U937 and U266 cells. Caspase-3, a member of the caspase family that plays a critical role in the process of the apoptotic program, is primarily responsible for the cleavage of PARP during cell death [42]. Procaspase-3 is reduced by SSD treatment. Our results also showed that SSD treatment markedly elevated the protein expression of the pro-apoptotic protein Bax. Accordingly, TUNEL-positive cells were increased in SSD-treated U266 and U937 cells, indicating that SSD induces apoptosis. 

The ER, an organelle related to Ca^2+^ storage and protein folding/maturation, plays a pivotal role in protein synthesis and transport. Furthermore, ER stress is associated with various diseases including inflammation, diabetes, and cancer [43]. ER stress-related proteins such as CHOP, ATF4, p-ATF2, PERK, and p-eIF2α are regulated by SSD treatment, demonstrating that SSD induces ER stress.

ROS formation is known to trigger misfolded or unfolded proteins, inducing ER stress and apoptosis [44,45]. To identify that ER stress induced by SSD was due to ROS generation, ROS was measured by a cellular reactive oxygen species detection assay kit. SSD treatment significantly increased ROS generation in U266 and U937 cells. NAC, an aminothiol and synthetic precursor of intracellular cysteine and GSH, has an important role as a ROS scavenger [46]. Therefore, NAC has been used in apoptosis research to investigate the role of ROS in the induction of apoptosis. Notably, the increased ROS generation and cytotoxicity induced by SSD was reversed by NAC pretreatment, indicating that SSD-induced apoptosis was due to ROS generation. U266 cells were more susceptible to SSD treatment than U937 cells. The difference might be based on a different ROS generation level and a different decreased level of miR-657 after SSD treatment.

MiR-657 is known by its oncogenic properties in several cancer types. It plays a critical role in lung cancer [47] and liver cancer [27,48]. Also, the overexpression of miR-657 has been found in larynx carcinoma [49] and metastasis of cervical squamous cancer cells [50]. However, the role of miR-657 in hematologic cancer has not yet been reported. We reported miRNA-related apoptosis brought on by some herbal medicines used to treat MM [51,52]. In this study, it was identified that treatment with SSD decreased the expression of miR-657 and the deregulation of miR-657 led to the inhibition of ER stress and apoptosis by the regulation of p-ATF2, CHOP, and PARP. However, when miR-657 mimic transfection was applied, cell viability was significantly increased and the apoptotic effect of SSD was attenuated via ER stress reduction. These results suggest that miR-657 may act as an onco-miRNA via ER stress regulation and that SSD could induce apoptosis by the inhibition of this onco-miRNA and ER stress induction. Taken together, this study reports for the first time that SSD could be a potent anti-hematologic cancer agent inducing apoptosis via the modification of miR-657/ER stress pathways.

## 4. Materials and Methods

### 4.1. Chemicals and Reagents

*Spatholobus suberectus* Dunn (200 g) was harvested in Hongchungun, Gangwondo, Korea. A voucher specimen (no. KH-00086) was stored at the herbarium of the Cancer Molecular Targeted Herbal Research Center of Kyung Hee University. The preparation and extraction were carried out as previously described [53,54]. Briefly, SSD was extracted twice in 100% ethanol (EtOH, 1 L × 2) for 3 days each. The extracted solutions were filtered and evaporated to produce an EtOH extract (9.4 g, percent yield = 4.7%).

### 4.2. HPLC Analysis

Chromatographic analysis was conducted with an Agilent series 1290 system (Agilent Technologies, Palo Alto, CA, USA) equipped with a quaternary pump, a vacuum degasser, an auto-sampler, and a reverse-phase packing C18 column (4.6 mm × 250 mm, 4 µm). The gradient elution was composed of solvent (A) (water:0.1% phosphoric acid, *v/v*) and solvent (B) (water:30% acetonitrile, *v/v*). The mobile condition was performed as follows: 0 min, 5% (B); 5 min, 5% (B); 6 min, 25% (B); 13 min, 35% (B); 15 min, 75% (B); 17 min, 95% (B); 21 min, 5% (B); 22 min, 3% (B). The flow rate was 0.8 mL/min and the injection volume was 10 µL. The thermostatted column was operated at 30 °C and a programmable various-wavelength UV-Vis detector was used at 250 nm, 280 nm, and 310 nm. All solutions were prepared with 1 µm filtration (Sigma-Aldrich, St. Louis, MO, USA) for the samples and 1.47 mm filtration (Sigma-Aldrich) for the eluent. The mobile phase was purged before injection onto HPLC. Epigallocatechin (EGC) (Sigma-Aldrich), genistein (Sigma-Aldrich), and SSD were accurately weighed and then dissolved in acetonitrile (ACN): water (30:70, *v/v*). Identification was achieved by comparing retention times and UV-wavelength.

### 4.3. Cell Culture

U266 (multiple myeloma cell line), THP-1 (acute monocytic leukemia cell line), and K562 (chronic myelogenous leukemia cell line) cells were purchased from American Type Culture Collection (ATCC, Manassas, VA, USA). U937 (myeloid leukemia cell line), CPAE (normal pulmonary artery endothelial cell line), CCD-18Co (normal colon epithelial cell line), and MDBK (normal kidney cell line) cells were obtained from KCLB (Korean Cell Line Bank, Seoul, Republic of Korea). The U266, U937, THP-1, K562, CPAE, and MDBK cells were cultured in RPMI1640 medium supplemented with 10% fetal bovine serum (FBS), 2 μM L-glutamine, and 10,000 U/mL penicillin/streptomycin (Gibco, Grand Island, NY, USA). DMEM with L-glutamine (300 mg/L), 25 mM HEPES, and 25 mM NaHCO3 (90%) or heat inactivated fetal bovine serum (10%) was used for CCD-18Co cells culture. Cells were incubated at 37 °C in a humidified atmosphere of 5% CO_2_. The cell medium was changed to reach 80–90% confluency every 2–4 days.

### 4.4. Cytotoxicity Assay

The cytotoxic effect of SSD against U266, U937, THP-1, K562, CPAE, CCD-18Co, and MDBK cells was assayed using an EZ-CYTOX cell viability assay kit (Daeil Lab Service, Seoul, Korea) according to the manufacturer’s instruction. Briefly, cells were seeded onto a 96-well microplate and treated with various concentrations of SSD, genistein, and EGC (0, 12.5, 25, 50, 100, 200, 400 μg/mL) for 24 h. To measure the optical density, a microplate reader (Bio-Rad, Hercules, CA, USA) was used at 450 nm. Cell viability was determined as a percentage of viable cells in the drug-treated group versus the untreated control.

### 4.5. Western Blot Analysis

Proteins from cell were lysed in RIPA buffer (pH = 7.4, 50 mM Tris-HCl, 150 mM NaCl, 1% NP-40, 0.25% sodium deoxycholic acid, 1 mM Na3 VO4, 1 M EDTA, 1 mM NaF) containing protease inhibitors cocktail (Amresco, Solon, OH, USA). The protein contents of samples were assayed using a Bio-Rad DC protein assay kit II (Bio-Rad, Hercules, CA, USA). Protein samples were separated by electrophoresis on SDS-PAGE 8%, 10% gels. After blocking in 5% non-fat milk, the membrane was incubated with primary antibodies for pro-PARP, pro-caspase-3, Bax, CHOP, p-eIF2α, ATF4, p-ATF2, PERK, and β-actin (Cell Signaling, Beverly, MA, USA) followed by the exposure to horseradish peroxidase (HRP)-conjugated secondary anti-mouse or rabbit antibodies (Bioss Antibodies, Woburn, MA, USA). To visualize protein bands, a chemiluminescence (ECL) system (Amersham Pharmacia, Piscataway, NJ, USA) was used.

### 4.6. TUNEL Assay

To detect cell death, a DeadEndTM Fluorometric terminal deoxynucleotidyl transferase-mediated dUTP-biotin nick end labeling (TUNEL) system kit was used according to the manufacturer’s instructions (Invitrogen, Carlsbad, CA, USA). In brief, U266 or U937 cells (2 × 10^5^ cells/mL) were treated with SSD for 24 h and plated onto a poly-L-lysine-coated slide. Cells were fixed with 4% paraformaldehyde for 30 min and washed twice with phosphate-buffered saline (PBS) for 2 min. Fixed cells were exposed to permeabilization solution (0.1% Triton X-100 and 0.1% Sodium citrate) and incubated with terminal deoxyribonucleotidyl transferase (TdT) enzyme buffer containing fluorescein-12-dUTP for 60 min at room temperature (RT) in the dark. The slides were mounted with mounting medium containing 4′,6-diamidino-2-phenylindole (DAPI) (VECTOR, Burlingame, CA, USA). The TUNEL-stained cells were visualized by FLUOVIEW FV10i confocal microscopy (Olympus, Tokyo, Japan).

### 4.7. Measurement of ROS

A Cellular Reactive Oxygen Species Detection Assay (Abcam, Cambridge, United Kingdom) with reagent 2′,7′-dichlorofluorescin diacetate (DCFDA) measures hydroxyl, peroxyl, and other ROS activity within the cells. 2′,7′-dichlorofluorescin (DCF) was used for the detection of cytosolic cellular hydrogen peroxide (H_2_O_2_) by fluorescence spectroscopy. U266 or U937 cells (2 × 10^5^ cells/mL) were pretreated with *N*-Acetyl-L-cysteine (NAC, Sigma Aldrich Co., St. Louis, MO, USA) for 2 h and treated with SSD (10 or 20 µg/mL) for 24 h. Then cells were stained with 20 μM DCFDA containing 1× buffer for 30–45 min at room temperature (RT) in the dark, then seeded onto a 96-well plate and measured using a microplate reader (Bio-Rad, Hercules, CA, USA) (E_x_/E_m_ = 485/535 nm).

### 4.8. Quantitative Real-Time PCR Analyses

RNA was isolated from cells using TRIzol reagent (Invitrogen, Carlsbad, CA, USA) and cleaned using a RNeasy Mini kit (Qiagen, Seoul, Republic of Korea). The total RNA was reverse transcribed using mRQ enzyme (Takara, Tokyo, Japan), performed on 1 μg of total RNA using Mir-X^TM^ miRNA First-Strand Synthesis and a SYBR qRT-PCR Kit (Takara) as per the manufacturer’s protocol. In brief, for the mature-miRNA reverse transcription of miRNA, 3′-Primer and U6 primers supplied by Mir-X^TM^ miRNA First-Strand Synthesis and SYBR^®^ qRT-PCR Kit (Takara) and an miRNA-specific 5′ sequence were applied by Bioneer (Bioneer Corporation, AccuTarget^TM^ Human miRNA Mimic and Inhibitor Library, Daejeon, Republic of Korea) with the following sequences: has-miR-657 5-forward-5′-GGCAGGUUCUCACCCUCUCUAGGATGAC-3′. PCR was initially performed at 95 °C for 10 s, 95 °C for 5 s, and 60 °C for 20 s, followed by 40 cycles at 95 °C for 60 s, 55 °C for 30 s, and 95 °C for 30 s. Relative miRNA changes in contents were normalized using the standard *C*_t_ level of U6 snRNA. Three individual miR-specific values were calculated and averaged to obtained means ± standard deviations (SDs). RT-qPCR was performed using a LightCycler^TM^ instrument (Roche Applied Sciences, Indianapolis, IN, USA).

### 4.9. Microarray

The total RNA quality and quantity were assessed by Agilent bioanalyzer 2100 analysis. Human microRNA expression was analyzed with a miRCURY LNA^TM^ microRNA array, 7th gen-has, mmu and rno array (EXIQON, Vedbaek, Denmark), covering 1918 well-characterized human microRNA among 3100 capture probes for human, mouse, and rat miRNAs. In this procedure, 5′-phosphates from 1 μg of total RNA was removed by treating Calf Intestinal Alkaline Phosphatase (CIP) followed by labeling with Hy3 green fluorescent dye. Labeled samples were subsequently hybridized by loading onto a microarray slide using a Hybridization Chamber Kit part # G2534A (Agilent Technologies, Santa Clara, CA, USA) and a Hybridization Gasket Slide Kit part # G2534-60003 (Agilent Technologies). Hybridization was performed over 16 h at 56 °C followed by washing the microarray slide as recommended by the manufacturer. Processed microarray slides were then scanned with an Agilent G2565CA Microarray Scanner System (Agilent Technologies, Santa Clara, CA, USA). Scanned images were imported by Agilent Feature Extraction software version 10.7.3.1 (Agilent Technologies) and the fluorescence intensities of each image were quantified using the modified Exiqon protocol and corresponding GAL files.

### 4.10. Data Analysis for Microarray

The data were analyzed with the quantile normalization method. The normalized and log-transformed intensity values were then analyzed using GeneSpring GX 13.1.1 (Agilent Technologies, Santa Clara, CA, USA). This normalization method aims to make the distribution of intensities for each array in a set of arrays the same. The normalized and log-transformed intensity values were then analyzed using GeneSpring GX 13.1.1 (Agilent Technologies, Santa Clara, CA, USA). Fold change filters included the requirement that the genes presented in at least 150% of controls for upregulated genes and less than 66% of controls for downregulated genes. In the Cluster 3.0 program, using the Euclidean distance and average linkage algorithm gave a hierarchical clustering analysis.

### 4.11. Statistical Analysis

Statistical analyses of the data were conducted using Sigmaplot^®^ version 12 software (Systat Software Inc., San Jose, CA, USA). All data were expressed as means ± standard deviations (SDs). The statistically significant differences between the control and treatment groups were calculated by the Student’s *t*-test and one-way ANOVA test.

## 5. Conclusions

The results indicate that SSD exerted cytotoxicity against U266, U937, THP-1, and K562 cancer cells while CPAE, CCD-18Co, and MDBK normal cells were less affected by SSD. The treatment of SSD facilitated ROS-dependent ER stress and triggered apoptosis by regulating CHOP, cleaved PARP, cleaved caspase-3, and Bax in both U266 and U937 cells. The mimic of miR-657 increased the viability of SSD-exposed U266 and U937 cells, indicating that miR-657 inhibition plays a role in SSD-induced apoptosis. Also, SSD treatment decreased the expression of miR-657, leading to the elevation of *p*-ATF2, CHOP, and cleaved PARP expression levels. In conclusion, SSD induced apoptosis via a novel miR-657/ATF2 signal pathway modification.

## Figures and Tables

**Table 1:** Measurement repeatability of intra-day precision and inter-day precision was performed against reference standards; the percentage of RSD of six assay results was calculated.

**Inter-day**				
	**Standards**		**SSD** ^1^	
	RT ^2^ (min)	RSD ^3^ (%)	RT ^2^ (min)	SD ^4^ (%)
EGC	2.412	0.078	2.376	0.172
Genistein	13.88	0.102	13.913	0.500
**Intra-day**				
	**Standards**		**SSD** ^1^	
	RT ^2^ (min)	RSD ^3^ (%)	RT ^2^ (min)	SD ^4^ (%)
EGC	2.411	0.017	2.381	0.689
Genistein	13.888	0.689	13.844	0.900

^1^ SSD: *Spatholobus suberectus* Dunn, ^2^ RT: retention time, ^3^ RSD: relative standard deviation, ^4^ SD: standard deviation.
